# Electroporation of mammalian cells by nanosecond electric field oscillations and its inhibition by the electric field reversal

**DOI:** 10.1038/srep13818

**Published:** 2015-09-08

**Authors:** Elena C. Gianulis, Jimo Lee, Chunqi Jiang, Shu Xiao, Bennet L. Ibey, Andrei G. Pakhomov

**Affiliations:** 1 Frank Reidy Research Center for Bioelectrics, Old Dominion University, Norfolk, VA 23508, USA; 2 Department of Electrical and Computer Engineering, Old Dominion University, Norfolk, VA 23508, USA; 3 Bioeffects Division, 711^th^ Human Performance Wing, Air Force Research Laboratory, Fort Sam Houston, San Antonio, TX 78234, USA

## Abstract

The present study compared electroporation efficiency of bipolar and unipolar nanosecond electric field oscillations (NEFO). Bipolar NEFO was a damped sine wave with 140 ns first phase duration at 50% height; the peak amplitude of phases 2–4 decreased to 35%, 12%, and 7% of the first phase. This waveform was rectified to produce unipolar NEFO by cutting off phases 2 and 4. Membrane permeabilization was quantified in CHO and GH3 cells by uptake of a membrane integrity marker dye YO-PRO-1 (YP) and by the membrane conductance increase measured by patch clamp. For treatments with 1–20 unipolar NEFO, at 9.6–24 kV/cm, 10 Hz, the rate and amount of YP uptake were consistently 2-3-fold higher than after bipolar NEFO treatments, despite delivering less energy. However, the threshold amplitude was about 7 kV/cm for both NEFO waveforms. A single 14.4 kV/cm unipolar NEFO caused a 1.5–2 times greater increase in membrane conductance (p < 0.05) than bipolar NEFO, along with a longer and less frequent recovery. The lower efficiency of bipolar NEFO was preserved in Ca^2+^-free conditions and thus cannot be explained by the reversal of electrophoretic flows of Ca^2+^. Instead, the data indicate that the electric field polarity reversals reduced the pore yield.

Permeabilization of biological and artificial membranes by high voltage electric pulses, or electroporation, has been well studied in recent decades^1^,^2^,^3^,^4^. The permeabilized state of the cell membrane may persist for minutes, thereby enabling such applications as intracellular delivery of drugs, plasmid DNA, and siRNA, as well as tissue and tumor ablation^5^,^6^,^7^,^8^,^9^. Decreasing the electric pulse duration into the nanosecond range engages different mechanisms of membrane potential build-up (dielectric stacking instead of Maxwell-Wagner polarization) and enables direct effects of the electric field on intracellular membranous structures^4^,^10^,^11^,^12^, thereby giving rise to specific biological effects and opening new opportunities to control cell function. Distinctive effects of nanosecond electric pulses include permeabilization of mitochondria and endoplasmic reticulum, as well as formation of long-lived nanometer-size membrane defects (“nanopores”), smaller than with longer micro- and millisecond pulse treatments^13^,^14^,^15^,^16^. These primary events initiate a horde of downstream physiological changes which may include cytosolic Ca^2+^ mobilization^12^,^17^,^18^,^19^,^20^,^21^, modulation of endogenous ion channels^22^,^23^, pyknosis^9^, cytoskeleton disassembly^24^,^25^,^26^, cytoplasm granulation^27^, cell swelling and blebbing^14^,^28^,^29^, and necrotic or apoptotic cell death^29^,^30^,^31^,^32^.

Recently we have reported another feature which is unique for nanosecond pulses and clearly separates them from micro- and millisecond stimuli. Specifically, we found that at least some of the above effects of nanosecond pulses can be prevented (or perhaps undone) by a prompt application of additional nanosecond electric pulses of the opposite polarity^33^. In other words, bioeffects which have already been initiated by the first electric pulse could be cancelled by an opposite polarity pulse, or just by reversing the electric field direction within the same pulse (i.e., by applying a bipolar pulse). The singularity of this “bipolar cancellation” phenomenon is emphasized by the fact that bipolar nanosecond pulses are far less efficient than unipolar pulses despite having a double duration and delivering twice more energy. This phenomenon contrasts the effects of micro- and millisecond-duration bipolar pulses, which usually are similarly or more effective than unipolar pulses of the same total duration^34^,^35^,^36^,^37^,^38^,^39^,^40^ (see Pakhomov *et al.*^33^ for a more detailed comparison).

Three mechanisms have been identified as potentially responsible for the bipolar cancellation^33^,^41^: (1) the assisted membrane discharge which shortens the time when membrane is above the critical breakdown potential, (2) poration as a two-step chemical process, where the first phase involves charge transfer and thus can be reverted by the electric field reversal, and (3) the primary role of the electrophoretic transport of charged species, and Ca^2+^ in particular, so that the electric field reversal may drive these species out of the cell and decrease the net effect. The feasibility of the latter mechanism has been confirmed by a theoretical analysis^41^. Below we report an experimental testing of this mechanism which, however, found that the bipolar cancelation does not necessarily rely on the reversal of direction of Ca^2+^ electrophoresis.

A concurrent aim of this study was to extend the observation of bipolar cancellation from individual rectangular pulses to a different type of stimuli, namely to nanosecond electric field oscillations (NEFO). An individual bipolar NEFO (Fig. 1A) is essentially a damped sine wave which can serve as a laboratory surrogate of high power electromagnetic pulse and ultra-wide band emissions, two environmental factors which have been intensely studied for physiological and health effects^42^,^43^,^44^,^45^,^46^. We hypothesized that the bipolar cancellation effect could underlie the experimentally established inefficiency of these emissions as biological stimuli. However, NEFO are substantially different from previously studied rectangular nanosecond pulses (by a slow risetime, the lack of a plateau at the high voltage, sharply reduced amplitude of the reverse polarity phase, and by a fast repetition of several polarity reversals), thus making extrapolation of the earlier findings to NEFO questionable. Finally, we also aimed to shed light on mechanisms underlying the bipolar cancellation by analyzing how the electroporation by bipolar NEFO and subsequent recovery depend on the electric field amplitude and delivery protocols. For comparison, we used a unipolar NEFO which had no polarity reversals to evoke the bipolar cancellation. The unipolar NEFO was produced by rectification of the bipolar one and therefore had essentially the same peak amplitude and duration (Fig. 1A).

## Results

### Electric field reversals reduce membrane permeabilization by NEFO

**Figure 1:**
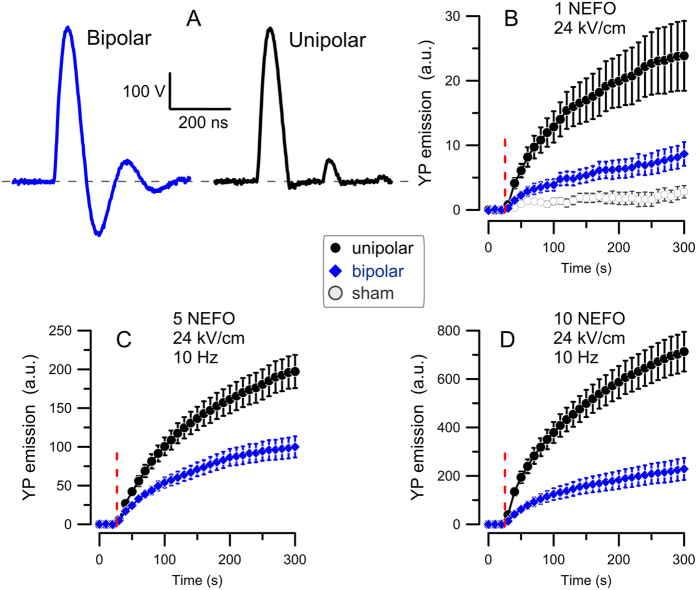
Unipolar and bipolar nanosecond electric field oscillations (**A**) have different potency to electroporate CHO cells (B–D). (**A**) The two types of NEFO have the same shape and amplitude of positive-going phases, with the first phase duration of 140 ns at 50% height. The unipolar NEFO lacks negative-going phases 2 and 4. (**B–D**) Electroporation is revealed by the time-lapse imaging of YP dye uptake. Cells were exposed to either 1 (**A**), 5 (**B**), or 10 (**D**) unipolar (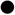) or bipolar (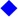) NEFO (24 kV/cm, 10 Hz) at 28 s into the experiment (vertical dashed line). The exposure parameters are also provided in panel legends. Mean ± SE for 9–23 cells in each group. Higher dye uptake in cells exposed to unipolar NEFO was significant in all groups (p < 0.01). A common control group subjected to sham exposure (plotted in panel (**B**) only) showed no appreciable fluorescence gain.

The uptake of fluorescent dyes which do not cross the intact plasma membrane is commonly employed to detect and quantify membrane permeabilization by electroporation and to study the time dynamics of resealing^4^,^13^,^14^,^47^. Among such dyes, YO-PRO-1 (YP) is a DNA stain which is practically non-fluorescent until entering the cell and binding to nucleic acids^48^. We used time-lapse imaging to monitor YP fluorescence every 10 s for 5 min. The first three images (baseline) were taken prior to the NEFO exposure which commenced at 28 s into the experiment. Figure 1B shows that even a single NEFO at 24 kV/cm (hereinafter the indicated electric field strength is the peak of the first phase), either bipolar or unipolar, immediately triggered sustained YP entry which continued, at a declining rate, until the end of the experiment. Sham-exposed cells (which underwent all the same procedures, but triggering of NEFO was disabled) showed no change in fluorescence. Applying trains of 5 and 10 pulses (Fig. 1C,D) expectedly evoked more and faster YP uptake, indicating a higher degree of membrane permeabilization. For all these conditions, the unipolar NEFO was consistently more efficient than the bipolar one, causing 2–3.5 times greater YP uptake. Since the only appreciable difference between the two NEFO waveforms was the presence of the negative-going second phase in the bipolar NEFO, we conclude that it was the reversal of the electric field that attenuated (canceled) the effects which were initiated by the first phase, similar to the earlier observations with rectangular nanosecond pulses^33^.

**Figure 2:**
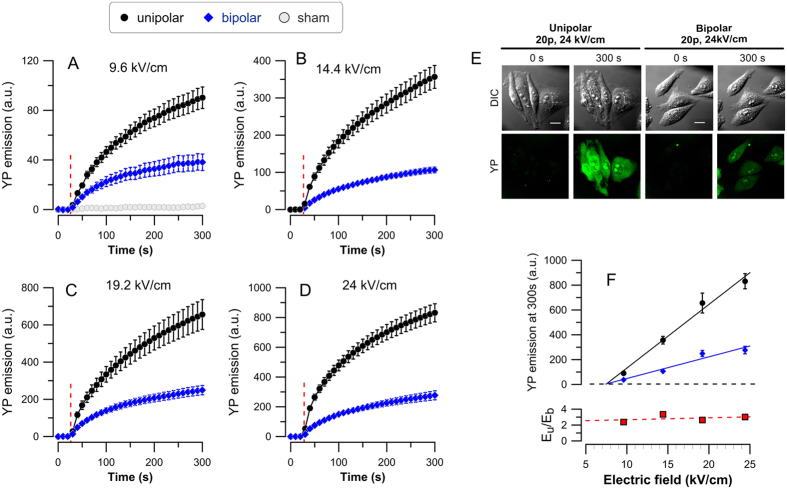
Increased electric field augments electroporation (A–D) but has little impact on the relative efficiency of uni- and bipolar NEFO (F). (**A–D**) YP dye uptake triggered by cell permeabilization with a train of 20 unipolar (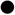) or bipolar (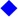) NEFO, delivered at 10 Hz starting at 28 s. The peak amplitude of NEFO is indicated in legends. Mean ± SE for 19–29 cells in each group. A control group subjected to sham exposure is plotted in (**D**) only. See Fig. 1 for more details. (**E**) Representative differential-interference contrast (DIC; top) and YP fluorescence images (bottom) before exposure and at 300 s after 20 unipolar or bipolar NEFO at 24 kV/cm, 10 Hz (left and right panels, respectively). Scale bars are 10 μm. Note greater YP uptake and morphological changes (swelling, blebbing, and cytoplasm granulation) after unipolar NEFO. (**F**) The effect of NEFO amplitude on YP emission reached by 300 s (top plot) and the ratio of emission values after uni- and bipolar NEFO (E_u_/E_b_, bottom). Both linear fits (top) cross the zero fluorescence level (dashed line) at ~7 kV/cm, indicating a common threshold for electroporation by either NEFO waveform. The unipolar NEFO caused 2–3 fold greater YP uptake with no apparent dependence on the peak electric field (bottom).

We further checked if the degree of cancellation can be controlled by the stimulus intensity. A brief 10 Hz train of 20 NEFO applied at 28 s into the experiment triggered immediate YP uptake at pulse amplitudes from 9.6 to 24 kV/cm (Fig. 2A–D), but not at 4.8 kV/cm (data not shown). Within the electroporating range of amplitudes, the dye uptake (as measured by the end of the experiment) increased linearly with increasing the electric field for both uni- and bipolar NEFO, whereas the latter consistently was 2–3 fold less effective (Fig. 2F). The linear fits for uni- and bipolar NEFO crossed zero at the same extrapolated electric field strength of 7 kV/cm, which can be regarded as a threshold for both waveform types. This value is remarkably close to 6 kV/cm electroporation threshold for 60-ns rectangular pulses, as measured by patch clamp^49^, arguably the most sensitive method to detect electroporation^50^. In contrast to long (micro- and millisecond) electric pulses, electroporation by NEFO may rely on dielectric stacking rather than Maxwell-Wagner polarization^50^ thus being less dependent on the cell size or shape^51^; hence the above measurements of the electroporation threshold can hold true for a variety of cells. The greater extent of cell membrane disruption by unipolar NEFO is also manifested by morphological changes (cell swelling, blebbing, and cytoplasm granulation), whereas cells exposed to bipolar NEFO showed little or no change (Fig. 2E).

**Figure 3:**
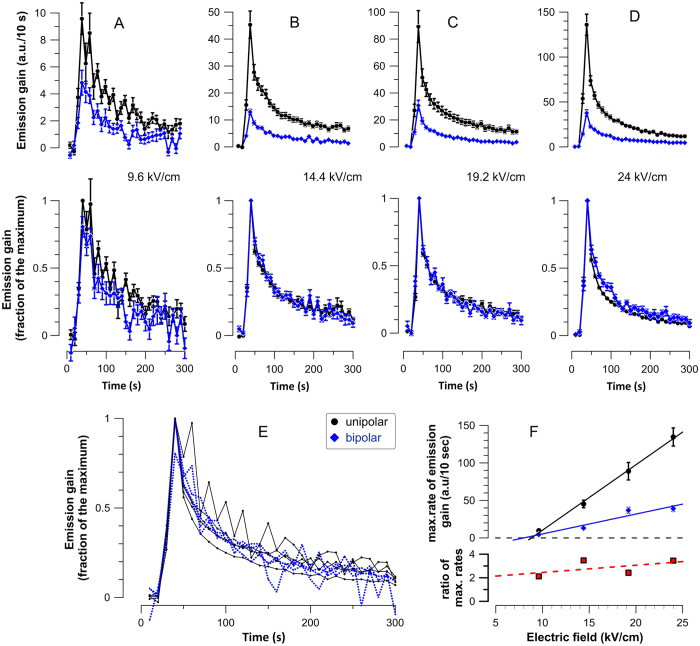
The resealing kinetics of electropermeabilized cells does not depend on the type of NEFO or its amplitude. Panels (**A–D**) (top row) show the change in YP fluorescence between the sequential images (i.e., emission gain per 10-s intervals). In the respective bottom row plots, the data are normalized to the maximum emission gain values for each plot. Note the essentially identical time course of the plots for uni- and bipolar NEFO. All data are from the experiments presented in Fig. 2. (E) All eight normalized curves from the above panels plotted together to show no apparent impact of either shape or amplitude of NEFO. Error bars are omitted for clarity. (**F**) The effect of NEFO amplitude on the maximum YP emission gain. See Fig. 2 and text for more details.

The rate of YP uptake gradually decreased with time after exposure, reflecting the shrinkage and/or resealing of NEFO-opened membrane pores (Fig. 3). This figure shows the difference in YP fluorescence between the sequential images (i.e., the gain in fluorescence per 10-s interval between the images). For all studied NEFO amplitudes, the maximum YP uptake rate was 2-3-fold lower for bipolar NEFO (Fig. 3F); however, the pore resealing kinetics for uni- and bipolar NEFO showed no difference. When the dye uptake rates were normalized to the peak value for the respective treatment, the dynamics of the rate reduction after both types of NEFO were the same (Fig. 3A–D, lower panels). Moreover, there was no difference in the rate reduction kinetics for NEFO applied at different electric field amplitudes (Fig. 3E). Consistent with this observation, fitting the reduction of the YP uptake rate after uni- and bipolar NEFO treatments with a double-exponential function showed no difference in either fast or slow time constants. For example, for 24 kV/cm uni- and bipolar NEFO, respectively, the fast time constants were 7.9 +/− 0.6 s and 7.6 +/− 1.5 s, and the slow time constants were 93 +/− 15 s and 97 +/− 21 s. The similarity of the pore resealing/shrinkage kinetics suggests that uni- and bipolar NEFO opened membrane pores with similar properties which sealed at the same rate. Therefore, the increased YP uptake after unipolar NEFO should be attributed to the increased number of membrane pores formed, rather than to the formation of qualitatively different (e.g., larger) pores.

### Reduced efficiency of bipolar NEFO is preserved in a Ca^2+^-free medium

**Figure 4:**
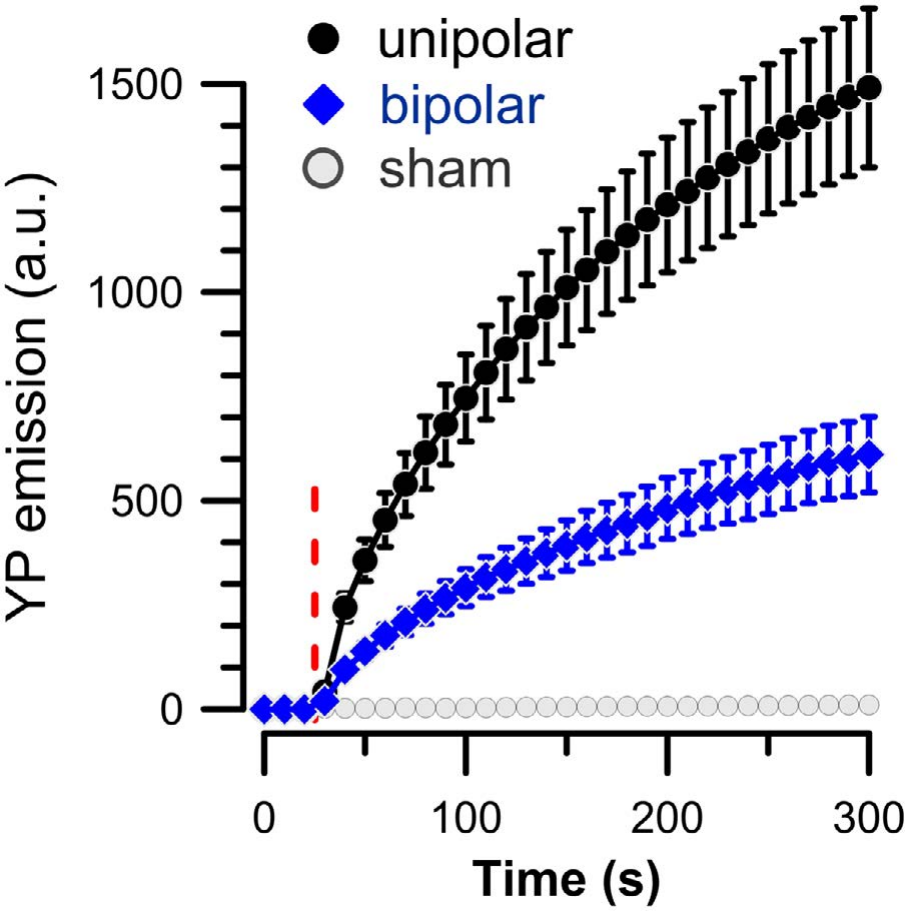
The reduced electroporation efficiency of bipolar NEFO is preserved in a Ca^2+^-free solution (no added Ca^2+^, 2 mM EGTA). Cells were exposed to a train of 20 unipolar (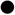) or bipolar (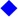) NEFO (10 Hz, 24 kV/cm), delivered starting at 28 s. Mean +/− SE for 12–17 cells in each group. See Fig. 1 for other details.

One of mechanistic hypotheses aimed at explaining the bipolar cancellation phenomenon points to the reversal of electrophoretic entry of Ca^2+^ when the electric field polarity is reversed^33^,^41^. This hypothesis considers various long-lasting effects of nanosecond pulses (such as YP uptake and cell death) as consequences of cell overload with Ca^2+^ during the electric pulse. In the case of a bipolar pulse, the first phase of the pulse facilitates the entry of Ca^2+^ into the electroporated cell, whereas the next phase moves it out and thereby decreases the net Ca^2+^ uptake and its consequences. To test this idea, we repeated the experiments shown in Fig. 2D in a Ca^2+^-free buffer. Contrary to theoretical predictions, unipolar NEFO still caused a 3-fold greater YP uptake (Fig. 4). Thus, the reversal of Ca^2+^ flow across the plasma membrane was ruled out as a reason for the reduced effect of bipolar stimuli. With that said, nanosecond stimuli can permeabilize the cytoplasmic reticulum to elevate the cytosolic Ca^2+^ level^12^,^18^,^51^, so the possible impact of the reverse Ca^2+^ drift across the reticulum membrane has not been excluded.

### Lower efficiency of bipolar NEFO is manifested by a smaller increase in membrane electrical conductance

Compared with dye uptake studies, the electrical conductance of the cell membrane is a more direct measure of permeabilization. In addition, the whole-cell patch clamp method enables the effective control of both extra- and intracellular milieus, so the impact of intracellular Ca^2+^ can be evaluated more accurately. Earlier studies have identified patch clamp as arguably the most sensitive approach for detecting nanoelectroporation^14^.

**Figure 5:**
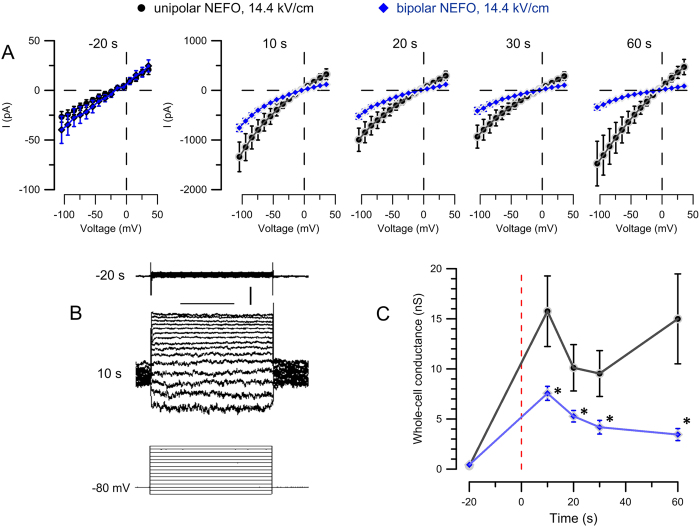
Reduced membrane permeabilization by bipolar NEFO is revealed by whole-cell electrical conductance measurements. (**A**) Current-voltage dependence in CHO cells at indicated times before (−20 s) and after exposure (10 to 60 s) to a single unipolar (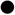) or bipolar (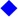) NEFO at 14.4 kV/cm. Note the different vertical scale in the first panel. (**B**) Traces of the whole-cell current in a representative cell, 20 s before (top) and 10 s after (center) the exposure to one bipolar NEFO. The bottom traces are the command voltage steps, from −100 to + 40 mV in 10-mV increments; the holding level is at −80 mV. Calibration bars: 100 μs and 250 pA. (**C**) The time course of the whole cell conductance in the experiments from panels (**A–E**). Mean ± SE for 11 cells in each group. Vertical dashed line denotes the time of NEFO application. Significant differences (p < 0.05) are labeled with asterisk in panel (**C**) only.

The first series of experiments (Fig. 5) was performed in the presence of 2 mM extracellular Ca^2+^, but using CHO cells which lack any voltage-gated Ca^2+^ channels. Pipette solution was buffered with EGTA, thereby excluding the impact of Ca^2+^ which could potentially be released from the endoplasmic reticulum^12^,^51^. The whole-cell configuration was established 1–2 min prior to the delivery of a single unipolar or bipolar NEFO at 14.4 kV/cm. The membrane conductance was measured in a voltage clamp mode, by applying a voltage-step protocol at 10 s prior to NEFO and then at 10, 20, 30, and 60 s after it.

Prior to the exposure, cells displayed almost linear current-voltage (I-V) dependence, with the average conductance of about 0.3 nS. At 10 s after NEFO, this value increased sharply, to 15.8 +/− 3.2 nS in cells exposed to unipolar NEFO, and to a smaller value of 7.6 +/− 0.8 nS (p < 0.05) in cells treated with bipolar NEFO. The I-V curves displayed slight inward rectification, which is typical for nanoporated cells^14^,^49^,^52^. The membrane pores gradually resealed, with nearly complete recovery by 60 s after the bipolar NEFO but not after the unipolar one. Instead, several cells developed a secondary increase in conductance, probably as a failure to recover after too severe electroporative damage. These cells lost the inward rectification, suggesting the breakdown of the nanopores into larger “conventional” electropores^52^ (data not shown).

**Figure 6:**
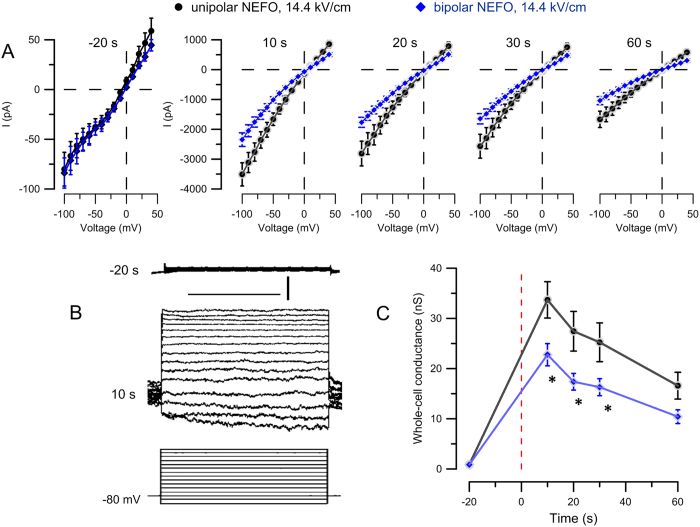
Reduced membrane permeabilization by bipolar NEFO is preserved in Ca^2+^-free conditions. (**A**) Current-voltage dependence in GH3 cells at indicated times before (−20 s) and after exposure (10 to 60 s) to a single unipolar (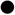; *n* = 10) or bipolar (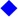; *n* = 12) NEFO at 14.4 kV/cm. Ca^2+^ was buffered with 2 mM EGTA in both intra- and extracellular solutions. Calibration bars in panel (**B**) are 100 μs and 500 pA. See Fig. 5 for other details.

In the second set of experiments (Fig. 6), we used GH3 cells to show that the above findings are not unique for CHO cells but can be reproduced across different cell lines. In order to fully exclude the impact of Ca^2+^, both the bath and pipette solutions were buffered with EGTA. Nonetheless, unipolar pulses were significantly more efficient at increasing the membrane conductance (p < 0.05 for all timepoints after exposure), although the difference from bipolar pulses was not as profound (1.5 times or less). These data prove incontrovertibly that the bipolar cancellation effect does not necessarily rely on Ca^2+^ drift.

## Summary

This study was the first to demonstrate that mammalian cells can be electroporated by damped sine wave electric stimuli of nanosecond duration. By comparing the efficiency of bipolar NEFO with a rectified (unipolar) NEFO of the same amplitude and duration, we established that the electric field polarity reversal hinders the electroporative efficiency of the bipolar NEFO. This phenomenon was likely a manifestation of the bipolar cancellation phenomenon which was described earlier for rectangular-shaped nanosecond pulses with the same amplitude of positive and negative phases^33^. Now, we observed that the second phase of only 35% of the first one is sufficient to partially cancel the effect of the first phase. It remains to be studied how the degree of cancellation depends on the ratio of the first and second phases (existing NEFO generators can only operate at a certain fixed ratio). Such studies may provide key information on how the biological efficiency of electric stimuli is controlled by shape and how it can be deliberately changed, e.g., for remote electrostimulation.

We also established that the bipolar cancellation phenomenon does not result from the reduced electrophoresis-driven Ca^2+^ entry into cells^41^. In diverse experiments, the bipolar cancellation was observed when free Ca^2+^ was buffered in the medium (Fig. 4), in the cytosol (Fig. 5), or in both (Fig. 6). Although under physiological conditions, Ca^2+^ entry will determine many downstream effects of electroporation^32^,^53^,^54^ and the reversal of the electric field may potentially reduce the net Ca^2+^ uptake^41^; here we report a direct and Ca^2+^-independent impact of the electric field reversal on the membrane permeabilization. While the underlying mechanism remains unknown, our data suggest that reversal of the electric field reduces the number of pores formed in the membrane without changing their size or resealing properties.

## Methods

### Cell culture, chemicals and solutions

Chinese hamster ovary (CHO-K1) cells and a murine pituitary cell line (GH3) were obtained from the American Type Culture Collection (ATCC, Manassas, VA) and maintained in culture at 37 °C, 5% CO_2_ per the supplier’s recommendations. CHO-K1 cells were cultured in Ham’s F12K medium supplemented with 10% fetal bovine serum (FBS, Atlanta Biologicals, Norcross, GA), 100 IU/mL penicillin, and 0.1 μg/mL streptomycin. GH3 cells were cultured in Ham’s F12K medium supplemented with 2.5% FBS, 15% horse serum (ATCC), 100 IU/mL penicillin, and 0.1 μg/mL streptomycin. The media and its components were purchased from Mediatech Cellgro (Herndon, VA). In the passage immediately preceding experiments, cells were transferred onto glass coverslips pretreated with poly-L-lysine to improve cell adhesion.

The composition of solutions utilized in different experiments is described in respective sections below. Chemicals were purchased from Sigma-Aldrich (St. Louis, MO) and Life Technologies (Grand Island, NY). The osmolality of the solutions was between 290 and 310 mOsm/kg, as measured by a freezing point microosmometer (Advanced Instruments, Inc., Norwood, MA). All experiments were performed at room temperature (22 ± 2 °C).

### Monopolar and Bipolar NEFO Exposure and Dosimetry

Two solid state pulse generators were designed and fabricated in-house to output a damped sine wave (referred to as bipolar NEFO) and a half-cycle sine wave (referred to as unipolar NEFO). Both pulse generators use IGBTs (IXLF19N250A) as the primary switch to obtain nanosecond duration voltage pulses, which are converted to sine-like waveforms at the output using self-wound transformers. For the half-cycle sine waveform output, the same circuit as the bipolar NEFO was used to first obtain a damped sine wave at the load. Then a diode in parallel to the secondary winding of the transformer was added to cut off the negative cycle (voltage) of the sine wave signal, thus a monopolar voltage was obtained at the load. Both pulse generators have the output impedance of 50 Ω, and are capable of delivering 560V maximum voltage at kilohertz repetition rates. Fluctuation of the peak voltage at a given input was less than 7% for both waveforms. Bipolar NEFO had 140 ns first phase duration at 50% height; the peak amplitude of phases 2–4 decreased to 35%, 12%, and 7% of the first phase. The unipolar NEFO did not include the negative-going phases 2 and 4 (Fig. 1A).

The procedures employed to expose cells to NEFO and to calculate the electric field were the same as described recently^13^,^25^,^33^,^55^. In brief, NEFO were delivered to a selected cell or group of cells with a pair of tungsten rod electrodes (0.1 mm diameter, 0.1 mm gap) upon a TTL trigger pulse from pClamp software via a Digidata 1322A output (Molecular Devices, Foster City, CA). The same software and Digidata output were used to synchronize image acquisition with NEFO exposure. The electrodes were positioned 30 μm above the coverslip so that the cells were located in the center of the gap between the electrodes. The electric field at the cell location was determined by 3D simulations with a finite-element Maxwell equation solver Amaze 3D (Field Precision, Albuquerque, NM). Cells were exposed to NEFO at 28 s after the onset of image acquisition sequence to allow for several baseline images to be taken prior to the exposure. The number of pulses and/or the peak amplitude were varied similarly for uni- and bipolar NEFO, in order to identify the specific impact of the electric field polarity reversals.

### Cell Imaging

Procedures for cell image acquisition and analysis were similar to those described previously^55^. Briefly, CHO-K1 cells plated on coverslips were placed in a glass-bottomed chamber mounted on an Olympus IX81 inverted microscope equipped with an FV1000 confocal laser scanning system. The chamber was filled with a physiological solution containing (in mM): 140 NaCl, 5 KCl, 2 CaCl_2_, 2 MgCl_2_, 10 HEPES, and 10 Glucose, pH to 7.4 with NaOH. For Ca^2+^-free conditions, CaCl_2_ was replaced with Na-EGTA. This solution was supplemented with 1 μM YP fluorescent dye which served as a marker of membrane permeabilization.

Differential interference contrast (DIC) and fluorescent images were obtained with a 40X, 0.94 NA dry objective. YP was excited at 488 nm, and emission was detected between 505 and 525 nm. Images were acquired every 10 seconds beginning before NEFO and continuing as a time series after it, and quantified using MetaMorph Advanced v.7.7.0.0 (Molecular Devices).

### Electrophysiology

Whole-cell patch clamp recordings were conducted similarly to what was previously described^49^,^52^. Recording pipettes were pulled from borosilicate glass (BF150-86-10, Sutter Instruments, Novato, CA) to a tip resistance of 1.5–3 MΩ using a Flaming/Brown P-97 puller (Sutter Instruments).

CHO-K1 or GH3 cells plated on coverslips were placed in the same microscope setup as was used for cell imaging. For experiments in CHO cells (Fig. 5), the chamber was filled with the same solution as for imaging (excluding YP dye); the pipette solution contained (in mM): 140 KCl, 5 K EGTA, 4 MgCl_2_, and 10 HEPES. pH was adjusted to 7.2 with KOH. For experiments in GH3 cells (Fig. 6), the bath and the pipette solutions were the same and contained (in mM): 140 Cs-Acetate, 5 K-EGTA, 4 MgCl_2_, and 10 HEPES; pH was adjusted to 7.2 with CsOH. This composition of the solutions was intended to eliminate any ion asymmetry across the membrane and to minimize currents through the endogenous voltage-gated channels, in order to isolate currents due to membrane electroporation.

Within 1–2 min after the whole-cell configuration was established, membrane currents were recorded at specific times before and after NEFO exposure by applying the same voltage-step protocol (200 ms steps from −100 to 40 mV in 10-mV increments); the holding potential between the sweeps was set at either −80 mV (for CHO-K1 cells) or 0 mV (for GH3 cells). Data were collected using a Multiclamp 700B amplifier, Digidata 1322A A-D converter, and pCLAMP 10 software (Molecular Devices). For CHO-K1 cell recordings, the command voltage was corrected offline for the junction potential of −4.5 mV. The whole-cell conductance (Figs. 5C and 6C) was calculated as chord conductance (i.e., as a slope of the straight line connecting the current at the most negative command voltage with the zero-current (reversal) potential on the voltage axis^56^) for each individual cell and then averaged across the group.

### Statistical Analysis

Data were presented as mean ± SEM for *n* number of cells per group (indicated in figure captions). Statistical analyses were performed using a two-tailed *t*-test where p < 0.05 was considered statistically significant.

## Additional Information

**How to cite this article**: Gianulis, E. C. *et al.* Electroporation of mammalian cells by nanosecond electric field oscillations and its inhibition by the electric field reversal. *Sci. Rep.* **5**, 13818; doi: 10.1038/srep13818 (2015).
